# Prevention and Recovery Care Services in Australia: Describing the Role and Function of Sub-Acute Recovery-Based Residential Mental Health Services in Victoria

**DOI:** 10.3389/fpsyt.2019.00735

**Published:** 2019-10-24

**Authors:** Justine Fletcher, Lisa Brophy, Helen Killaspy, Priscilla Ennals, Bridget Hamilton, Laura Collister, Teresa Hall, Carol Harvey

**Affiliations:** ^1^Centre for Mental Health, Melbourne School of Population and Global Health, The University of Melbourne, Parkville, VIC, Australia; ^2^Mind Australia Ltd, Heidelberg, VIC, Australia; ^3^School of Allied Health, Human Services and Sport, LaTrobe University, Bundoora, VIC, Australia; ^4^Division of Psychiatry, University College London, London, United Kingdom; ^5^Neami National, Preston, VIC, Australia; ^6^School of Nursing, Faculty of Medicine, Dentistry, and Health Sciences, The University of Melbourne, Parkville, VIC, Australia; ^7^Wellways, Fairfield, VIC, Australia; ^8^Nossal Institute for Global Health, Melbourne School of Population and Global Health, The University of Melbourne, Parkville, VIC, Australia; ^9^Department of Psychiatry, The University of Melbourne, Parkville, VIC, Australia; ^10^Psychosocial Research Centre, NorthWestern Mental Health, Coburg, VIC, Australia

**Keywords:** sub-acute, community-based residential environment, mental health, implementation, service delivery, built environment

## Abstract

**Background:** Prevention and Recovery Care (PARC) services are relatively new sub-acute residential services that have supported people with mental ill-health in Victoria since 2003. Operated from a partnership model between non-governmental agencies and clinical mental health services, PARC services integrate intensive recovery-focused psychosocial input with clinical mental health care.

**Aim:** To describe and contrast the 19 PARC services operating in Victoria at the time of the study, in terms of structures and function, resources, and content and quality of care.

**Method:** Nineteen participants, one representing each PARC, completed two surveys: the first, a purpose-designed survey relating to the government guidelines for PARC services, and the second, the Quality Indicator for Rehabilitative Care.

**Results:** Descriptive analyses highlighted that PARC services have operated in inner-city, urban, and regional areas of Victoria, from between 1 and 14 years. Participants reported that a recovery approach was at the core of service delivery, with a vast array of group and individual programs on offer. Across the state, there was variation in the quality of services according to the Quality Indicator for Rehabilitative Care domains.

**Conclusions:** This study has identified that there is variation in the structure and function, resourcing, and content and quality of care offered across Victoria’s PARC services even though, in the main, they are guided by government guidelines. Hence it appears that the services adapt to local needs and changes in service systems occurring over time. The findings indicate emerging evidence that PARCs are providing recovery-oriented services, which offer consumers autonomy and social inclusion, and therefore likely enable a positive consumer experience. The range of individual and group programs is in line with the Victorian guidelines, offering practical assistance, therapeutic activities, and socialization opportunities consistent with consumer preferences. Further research into implementation processes and their impacts on quality of care is warranted concerning this and similar service models.

## Introduction

Acute inpatient mental health care has been criticized for being expensive, restrictive, coercive, and unpopular with service users (1), and community-based residential alternatives have developed as a result. Acute inpatient care typically provides more intensive support to people experiencing a mental health crisis and/or a significant exacerbation of the symptoms of their mental illness requiring immediate treatment, although some community-based crisis services also exist for the same purpose. Slade et al. (2) compared inpatient and community-based alternatives, such as residential crisis services, and found no difference in outcomes but higher costs for community-based alternatives due to longer stays. Sweeney et al. (3) found that service users preferred crisis houses (a UK alternative for people who do not require involuntary hospital admission), due to stronger therapeutic relationships with staff, greater informal peer support, and fewer negative events experienced, for example, verbal abuse, forced medication, and being ignored by staff.

Aside from the aforementioned community-based crisis services, most community-based residential services are bed-based services that focus on improving the independence and community functioning of people with mental disorders (4). It is common to classify these services into sub-acute and non-acute services. One major difference between the residential service types is the length of stay. Non-acute services include community care units (CCUs) and residential rehabilitation services, which generally provide support for between 6 and 24 months (4). Operating since 2003, Prevention and Recovery Care (PARC) services are now a feature in most areas in Victoria, offering short-term support spanning from a few days to 4 weeks. PARC services are residential sub-acute services that support people with mental ill-health to either avoid an inpatient hospital admission (step-up) or leave hospital early (step-down). PARC services are now being implemented elsewhere in the country, with the aim of improving mental health outcomes and preventing hospital admissions for people who are acutely unwell (5). They have a strong emphasis on integrating clinical mental health care with intensive recovery-focused psychosocial input.

PARC services are considered part of the clinical system, that is, area mental health services (AMHSs) (state-funded specialist public mental health services, commonly described as clinical services). Elements of the PARC model of care are sub-contracted by the clinical service to a Mental Health Community Support Service (MHCSS) (non-government organization, NGO). This arrangement means the model of care is variable between PARCs, as they typically reflect the goals and needs of the local area as they are understood through the prism of the clinical provider. In practice, however, the relationship between the clinical service and MHCSS is collaborative and based on a shared commitment to the delivery of recovery-oriented sub-acute care. They are staffed by employees of both service types. Victoria’s adult sub-acute PARC services enable people to be admitted voluntarily, with or without a community treatment order, for up to 28 days. PARC services usually have a maximum of 10 residents, offering a homelike environment with single rooms in a stand-alone building. Thus, they are at least half the size of Victoria’s busy acute inpatient units that tend to be wards integrated into general hospitals or large health services. They contrast with other residential rehabilitation services because of the much shorter length of stay and emphasis on offering a residential support option as either a “step-up” from the community or a “step-down” from the inpatient unit. They are generally described as providing “sub-acute” care focused less on immediate treatment and more on recovery and social inclusion outcomes.

### Early Research Evidence About Alternatives to Admission

Since 2003, adult PARCs have gradually become firmly embedded in the area mental health service system in Victoria, and other Australian states have begun to adopt them. Despite considerable financial commitment and plans for expansion, the evidence base underpinning PARCs is very limited, largely relying on small, localized evaluations that, with rare exceptions (6), have not involved comparison groups, considered longer-term outcomes, or been published in the peer-reviewed literature (7–11). It remains unclear whether PARC services reduce pressure on acute beds in inpatient services (12), with only limited evidence available (13). A recent review of controlled studies concluded that current research is insufficient to provide convincing evidence about the effectiveness of residential alternatives to standard acute inpatient mental health services (14). This has led to calls for rigorous research to elucidate the models under which these services operate and their impact on stakeholders (9).

Even so, evaluations of Victorian PARC services and their equivalents in other Australian states do suggest they are well regarded by consumers. We use the term “consumer,” as it is common to move away from the language of “patient” when referring to people in such residential alternative services. In the Australian context of this study, we refer to “consumers,” akin to “service users.” In particular, consumers value services that are staffed by supportive and caring workers and offer practical assistance, therapeutic activities, and socialization opportunities (8, 11, 15). They have also been shown to be associated with improvements on recovery-related indicators such as role functioning (11, 15) and symptom-based measures (11).

The Victorian Department of Health (now the Department of Health and Human Services) PARC service guidelines were developed in 2010 with the goal of providing operational guidance regarding service planning and delivery (16). The government guidelines provide a framework for collaborative care planning that emphasizes rehabilitation and recovery that is adaptable to local need, enabling service provision that matches individual presentations and PARC resources. **Box 1** highlights the core components of the guidelines, including key service principles, service models, service operations, and performance monitoring. The current study is one component of a state-wide evaluation of PARC services. A series of interrelated studies have been designed with the principal aim of evaluating the appropriateness, effectiveness, and efficiency of adult PARC services in Victoria to address the gaps in knowledge regarding sub-acute community mental health residential services. This paper aims to describe and contrast the current PARC services operating in Victoria, in terms of structures and function, resources, and content and quality of care.

Box 1 **Summary of Prevention and Recovery Care Service guidelines.**

Key service principles

1. Collaboration
2. Least possible restrictive practices
3. Respect and responding to diversity
4. Consumer and carer participation
5. Privacy and confidentiality

Service model

- Active clinical community intervention and treatment
- Appropriate clinical treatment and support
- Appropriate range of types and levels of psychosocial and other support
- Active consumer involvement in their own treatment; maintaining and engaging consumers’ natural supports
- Client eligibility criteria, for example, consumers who are 16–64 years of age
- PARC services fit in the continuum of care between acute inpatient and intensive community support in the consumer’s own home; consideration should be given to the following
  - Entry processes
  - Care planning and implementation
  - Length of stay
  - Transfer of care/discharge planning
- Relationships
  - Links with clinical mental health services
  - Links with the community mental health support service
  - Links with primary and community-based services

*Governance:* appropriate agreements to be developed by the clinical service and the mental health community support service

Service operations

- Service planning and development consultation with a range of key stakeholders
- Staffing model and workforce development, for example, an appropriate mix of clinical and non-government organization (NGO) staff
- Daytime operations
- Nighttime operations
- Medication administration
- Incident management
- Clinical mental health and community mental health support service communication mechanisms
- Adherence to service standards and quality related to the Mental Health Act
- Complaints
- Additional policies and procedures, for example, medico-legal issues or sexual safety
- Catchment areas
- Facilities, location, and living environment

Performance monitoring

- Use of state-wide data collection and reporting systems
- Locally relevant measures

Summarized From the Adult PARC Services Framework and Operational Guidelines (16)

## Method

### Study Setting

As of January 2016, there were 19 adult PARC services offering approximately 184 beds in Victoria. By 2019, this increased to 20, with now only one AMHS in Victoria operating without a PARC service. These 19 PARC services open at the time this study was undertaken are sub-acute services, including a women’s-only service, that offer a 28-day maximum stay. The 19 PARC services included in this project include 12 PARC services in suburban areas, 4 in regional areas, and 3 in inner-city areas. Generally, the day-to-day management of the unit and the provision of psychosocial interventions and support is the responsibility of the MHCSS. The clinical services provide clinical governance and treatment and the assessment and management of risk issues in relation to individual consumers. The clinical services generally make decisions regarding entry and exit from the service in consultation with the MHCSS. The two services work in close partnership to provide an integrated and holistic approach to care.

### Participants

Each of the adult PARC services nominated a manager or other appropriately knowledgeable senior staff member to participate in the study (n = 19 participants).

### Data Sources

Two data sources were utilized:

1. Data on the PARC services were collected using a pro forma designed by the research team to reflect the alignment of services provided by each PARC service with the government guidelines and to provide details about the types of services offered (16). It included 37 quantitative items and open-ended items that generated qualitative data, and took approximately 20 min to complete. The following topics were covered: governance and operation; facilities, location, and living environment; service delivery; staff; key performance indicators (KPIs); and performance monitoring processes. **Table 1** provides examples of questions covering these topics.
2. The Quality Indicator for Rehabilitative Care (QuIRC) is an internationally validated tool designed for use in longer-term inpatient and community-based mental health residential facilities to assess the quality of care (17, 18). The QuIRC was considered suitable for this study because it is a validated instrument designed to measure the quality of care in a residential mental health setting. However, given that PARC services aim for a short length of stay, some adaptations were made (e.g., items referring to care provided over a 12-month period were changed to refer to a 1-month period). The QuIRC was designed for completion by the service manager and took around 1 h. It comprises 145 items that provide a combination of descriptive data and data that are collated into percentage scores on seven domains of care, with higher scores reflecting better quality on that domain. **Table 2** provides a brief description of the seven QuIRC domains. Because of the large number of items, it is not feasible to include its whole content; however, **Table 3** details the areas the QuIRC covers; see also (18).

**Table 1:** Example questions from the questionnaire concerning the alignment of services provided by each Prevention and Recovery Care (PARC) service with the government guidelines.

Topic	Example question	Response options
**PARC service governance and operational information**	Is there an operational collaboration agreement between the Adult Mental Health Service (AMHS) and the Mental Health Community Support Service (MHCSS)?	Yes/no
	What is the leadership structure in your PARC? Please comment on both the MHCSS leadership and AMHS leadership, and who has overall responsibility.	Open ended
**PARC service facilities, location, and living environment**	Which of the following best describes the type of location of the PARC service?	Co-location with MHCSS or community care unit (CCU)/single facility in the community/single facility on hospital grounds/cluster of closely linked facilities in the community/other (please specify)
**Service delivery**	Is there a particular model of practice that guides your PARC service delivery? If yes, please describe.	Yes/no and free text box
	Please describe the group programs that are offered to consumers.	Open ended
**Staff**	Please provide details about the MHCSS staff working at the PARC; number of staff and total equivalent full-time.	Open ended
**Key performance indicators and performance monitoring**	What locally relevant outcome measures do you use? Include consumer and carer outcome measures, quality improvement measures, exit and satisfaction surveys, or any other relevant measures.	Open ended

**Table 2:** The Quality Indicator for Rehabilitative Care (QuIRC).

Assesses 7 domains of care:	QuIRC 143 items assessing:
• Living (built) environment • Therapeutic environment • Treatments and interventions • Self-management and autonomy • Social interface • Human rights • Recovery-based practice	• Staffing, training, supervision • Built environment/facilities • Evidence-based interventions • Activities (in and outside the service) • Care planning processes • Service user involvement • Family support • Promotion of autonomy and independent living skills • Physical health promotion • Management of challenging behaviors • Complaints processes, confidentiality, access to advocacy and lawyer

**Table 3:** Description of QuIRC domains.

Domain	Description
Living environment	The built environment and the practical aspects of how the facility is organized and run
Therapeutic environment	The therapeutic culture of the facility, including staffing, training and supervision, staff attitudes to service users
Treatments and interventions	Medical, psychological, and social treatments and interventions, physical health promotion, and the use of seclusion or restraint
Self-management and autonomy	The degree to which the service assists service users to gain/regain skills for living independently
Social interface	The degree to which the service makes links with community resources and engages with service users’ families and carers to strengthen their social networks
Human rights	The degree to which service users’ legal and civic rights are promoted and how they are involved in decision making about their care; includes data protection, confidentiality and provision of systems for complaints, access to advocacy, and support with voting
Recovery-based practice	Degree to which service users are engaged collaboratively in planning and agreeing to their own care and treatment and in the running/decision making of the service; degree to which staff hold hope for service users to progress in their recovery

### Data Collection

The research team convened a forum in Melbourne in March 2017 for the senior staff participants to complete the Victorian PARC service mapping questionnaire and the QuIRC. The manager of each PARC service was sent a letter from the project team explaining the project and the required information to complete the audit tools at the forum. The nominated staff member was provided with the plain language statement and a consent form prior to the forum.

At the forum, each staff member was provided with an iPad to complete the audit tools, and members of the research team were available to clarify any questions that arose. Two service managers were unable to attend, so a researcher visited them to administer the survey tools within 1 month of the forum.

Ethics approval for this project was granted from the University of Melbourne’s Human Research Ethics Committee (project number: 1647880.1).

### Data Analysis

Quantitative data from the Victorian PARC service mapping questionnaire and the QuIRC were analyzed using SPSS Version 22 to generate descriptive statistics. Thematic analysis of qualitative data derived from open-ended questions regarding the types of individual and group programs described in the Victorian PARC service mapping questionnaire was undertaken by four of the authors. Initially, authors JF and LB discussed the qualitative content from the surveys and developed key themes to describe the data. JF then coded all the data under these themes. The themes and coding were then reviewed by JF, LB, CH, and BH, who discussed and negotiated the themes and coding until all parties were in agreement.

## Results

The first PARC service was established in Victoria in 2003, and the most recent one opened in 2016. On average, the PARC services had been operational for 7 years (SD = 7.3). All PARC services were staffed 24 h a day, 3 had a staff member awake and on duty at night, and the remaining 16 had a staff member in the building, sleeping over at night. PARC managers reported that their services had a mean of 10 beds (SD = 1, range 6–10), with a maximum length of stay of 28 days. Three PARC services had day places available, enabling a consumer to attend activities at the PARC during the day only, with two PARC services reporting that day places were used on average once per month.

### Victorian PARC Services Survey

#### Location, Building Type, and Access

The PARC services were located across the state in inner-city (3, 16%), suburban (12, 63%) and regional areas (4, 21%). Most were stand-alone facilities in the community (11, 58%). Others were co-located with MHCSSs or residential CCUs (4, 21%); two managers reported being in a cluster of closely linked facilities in the community, and two reported “other” location arrangements (none of the Victorian PARC services were on hospital grounds). Some PARC services were purpose-built facilities (11, 58%), while others operated from converted buildings (8, 42%). Managers of 10 of the 19 PARC services reported that the street entrance to the PARC was locked, and of these, the consumers of four, and staff of three, services were dependent on staff in the building granting access. This means consumers were free to come and go from the PARC service as they wanted, but for security in the community setting, the front doors were kept locked.

#### Staff Qualifications and Staffing Mix

**Table 4** displays qualifications of workers employed by the MHCSS, including the level of higher education and the details of staff with lived experience of mental health issues. Almost all staff (95%) were educated to graduate level and two-thirds to post-graduate master’s level. Most (84%) had a diploma in mental health. About half of the PARC services reported employing a peer worker, and two reported employing a family/carer peer support worker.

**Table 4:** Qualification of PARC staff employed by the MHCSS, staff mix between MHCSS and AMHS.

	Frequency	Percent		
**Staff qualifications**				
Diploma in mental health	16	84		
Diploma in alcohol and other drugs	10	53		
Diploma in clinical services	1	5		
Bachelor’s degree	18	95		
Master’s degree or higher	12	63		
Peer worker	11	58		
Consumer consultant	0	0		
Carer consultant	0	0		
Family and carer peer support worker	2	11		
Other (please specify)	5	26		
	**N of PARCs**	**Mean**	**SD**	**Range**
**MHCSS staff**				
Number of staff	19	10.4	2.9	4–16
Total equivalent full-time (EFT)	18^	7.3	1.4	4–10
**AMHS staff**				
Number of staff	19	5.6	3.9	1–15
Total EFT	18	2.6	1.6	1–8
**Other staff**				
Number of staff	19	0.2	0.5	0–2
Total EFT	19	0.4	1.3	0–4

^^^The data for one PARC was entered in error and so was removed from analysis.

The staffing mix of AMHS and MHCSS staff varied between PARC services. Most (17 of the 19 PARC services) had a permanent clinical staff member, and 6 had a system for the rotation of clinical staff (a planned length of time a clinical staff member would be assigned to work at the PARC). However, there was considerable variation in the percentage of time in a 24 h period that the clinical staff member was available (mean 44%, SD 28%, range 8–100%) and how much time in a 24 h period clinical staff were present (mean 32%, SD 20%, range 3–95%). **Table 4** shows the number and equivalent full-time (EFT) staff from the MHCSS, AMHS, and other services, that is, staff employed by outside agencies who work at the PARC.

#### Partnership Approach and Governance

Managers reported on the governance and operational procedures of the PARC services according to specific questions linked to government guidelines. Eighteen of the 19 PARC services had an operational collaboration agreement between the MHCSS and the AMHS, as well as a documented governance structure. Seventeen also had a sub-contract agreement for services to be delivered by the MHCSS. **Table 5** displays the policies each PARC service was expected to utilize according to the government guidelines and indicates the origin of each of the policies for PARC services.

**Table 5:** Types of policies and where they originate.

Policy	Joint		AMHS		MHCSS		N/A	
	N	Percent	n	Percent	n	Percent	n	Percent
Guidelines for entry and exit	15	78.9	3	15.8			1	5.3
Procedural document for admission and discharge	13	68.4	5	26.3			1	5.3
Risk assessment protocols	7	36.8	12	63.2				
Critical incidents policy	14	73.7	4	21.1	1	5.3		
Staff education and training policy	7	36.8	1	5.3	9	47.4	1	5.3
Complaints policy	9	47.4			9	47.4	1	5.3
Model of staff structure	10	52.6	1	5.3	7	36.8	1	5.3

Most PARC services had developed joint policies, particularly in relation to the day-to-day running of the PARC service, such as: guidelines for entry and exit; procedural documents for admission and discharge; and critical incidents. The risk assessment protocols were usually taken directly from the AMHS, whereas the staff education and training policy and the complaints policy were developed more often by the MHCSS.

#### Service Delivery

All referred to their approach to service delivery in terms of “recovery.” The “collaborative recovery model” (19) was the most frequently reported model (5, 26%). Other terms used to describe the approach to service delivery included “client-centered recovery framework,” “community recovery model,” “Recovery Star,” and “Mind’s recovery-oriented practice.” One PARC service described their service as using a “biopsychosocial model,” and two managers reported using the Victorian guidelines to support the approach to service delivery.

##### Group Programs

PARC service managers were asked the open-ended question, “Please describe the group programs that are offered to consumers.” Three managers included in their response that programming for groups was dependent on the needs and preferences of the consumers in the PARC service at the time. The following quote illustrates this point:

*We have a program whereby we ask the participants daily what types of things they would like to learn about. We then put in place groups that are relevant to the specific mix of participants that are in at the time. We have over the journey seen trends on what people are requesting and have got some resources that are used commonly.*

Another manager elaborated on how the service arranged groups:

*There is an extensive group plan that is set over two weeks and then rotates. The program is reviewed every six months to include feedback clients have provided either to staff, via the Peer Support Worker, or using the feedback forms at the end of their stay.*

Seventeen participants answered this by listing the array of programs and topics included in the PARC service group programs. Six interrelated and interdependent themes emerged from the data and are described below. **Table 6** shows the number of PARC services delivering groups within each theme and the volume of activities offered under each theme across all PARC services. The six themes describing group programs were:

**Table 6:** Delivery of groups and activities by theme.

Theme	Number of PARCs delivering, n = 17	Number of activity types offered across all PARCs	
		Total number	Range
Recovery and wellness	17	36	0 – 5
Activities of daily living (ADLs) and self-management	14	27	0 – 4
Physical health	12	19	0 – 3
Psycho-therapeutic interventions	12	17	0 – 3
Therapeutic milieu and group programs	11	23	0 – 3
Social groups	8	11	0 – 3

*Recovery and wellness:* This theme included recovery groups facilitated by peer workers; the Optimal Health Program (20); spiritual well-being; meditation and relaxation; and wellness planning. All 17 managers reported that their services offered groups under this theme. About half reported that their recovery groups were run by peers, and approximately half reported running relaxation groups.

*Activities of daily living (ADLs) and self-management:* This theme included the subthemes of self-care, cooking, and budgeting. All service managers reported running groups of various types within this theme, and almost all reported that their services offered a cooking group.

*Physical health* included nutrition and exercise. Approximately one-third of participants reported offering sport and recreation groups such as gym, swimming, and walking groups.

*Psycho-therapeutic interventions* represents interventions focused on consumers understanding and discovering strategies to ameliorate symptoms of mental ill-health. This theme included mindfulness groups, psychoeducation groups, and sensory groups. Twelve PARC managers reported that their service offered these kinds of groups. Mindfulness groups and psychoeducation groups were the most frequently reported (approximately half and one-third, respectively).

*Therapeutic milieu and activities:* This theme involved group activities, spanning the following areas: music; art/craft; yoga; dancing; community meetings; men’s group; and gardening. Eleven managers reported offering groups that were classified under this theme. Around half reported facilitating art and craft groups, and one-third reported running music groups and/or community meetings.

*Social groups* were defined as activities in the community supported by the PARC staff, including community connections, such as volunteering at a local animal shelter, and social outings, for example, to have afternoon tea at a local cafe. About half the participants reported facilitating social groups in the community. Each PARC seemed to focus on a particular type of outing, and there was not much commonality on the type of outings across the PARC services.

##### Individual Programs

All 19 participants provided details about the 1:1 programs offered to consumers. Many mentioned topics that related to the six themes identified for the group programs above, particularly concerning *recovery and wellness; ADLs and self-management;* and *physical health*. In addition, over half the participants reported 1:1 key worker support, and individual recovery planning, goal setting, and safety/wellness planning. Just under half of the services offered psychiatric and medical services, for example, psychiatric review, medication reviews and education, and referrals for counseling or therapy outside the PARC. Just under half of the participants reported that consumers had access to individual sessions based around the Optimal Health Program (20). One-quarter of participants reported that their consumers were offered sessions with a peer support worker and had access to family sessions and support.

#### Satisfaction and Experience of Service Measures

Exit surveys for consumers were in use in almost all PARC services, and about one-third used exit surveys with carers. Roughly three-quarters reported using clinical outcome tools (such as Health of the Nation Outcome Scales (HoNOS) or Behaviour and Symptom Identification Scale (BASIS-32)), and a minority reported the use of a recovery outcome measure.

#### Key Performance Indicators

Participants were asked to report KPIs (outlined in the government guidelines) for the previous 6-month period (1 July 2016–31 December 2016). **Table 7** details these. The average length of stay and occupancy rate were variable. Fourteen of the 19 PARC services sometimes included consumers who were subject to a community treatment order (orders under Victorian mental health legislation that can impose compulsory treatment in the community).

**Table 7:** Manager reports of key performance indicators as set out in the Victorian PARCs guidelines.

	N	Mean	Std. Deviation	Minimum	Maximum
What is the average length of stay in the PARC (in days)?	19	18.05	5.642	10	37
What has been your average occupancy rate? (%)	19	73.11	24.39	8	95
What is the average number of step-up admissions in a month?	19	15.58	21.269	2	70
What is the average number of step-down admissions in a month?	19	12.63	13.937	2	50
What is the average number of consumers on community treatment orders (CTOs) in a month?	19	1.74	1.727	0	6
What is the average number of consumers discharged from their CTO during their admission in a month?	19	0.63	1.165	0	5

### QuIRC

**Table 8** shows the overall descriptive statistics for each domain, as well as the percentage scores for each PARC service on the seven domains of the QuIRC. Services shown in blue in the body of the table were below the overall mean domain score. In the final column, green indicates the services with the least number of domains (0–2) scoring below the Victorian mean, amber indicates those in the mid-range (3–4), and red indicates those with the most domains (5–6) scoring below the Victorian mean.

**Table 8:** QuIRC domains by PARC.

PARC	Renovated or purpose-built	Living environment	Therapeutic environment	Self-management and autonomy	Social interface	Human rights	Treatments and interventions	Recovery-based practice	Number of domains below the Victorian average
04	PB	82	61.94	80.62	54.66	82.68	61.41	72.05	3
09	PB	80	62.64	64.94	66.76	73.04	60.12	65.89	4
13	PB	80	61.4	71.59	64.36	59.59	53.57	63.02	6
17	PB	88	56.43	65.36	67.41	70.76	52.58	57.16	5
02	PB	76	63.25	71.14	74.37	71.18	52.49	71.82	2
15	PB	66	63.74	70.53	55.08	70.12	71.75	63.26	5
19	PB	78	60.74	74.88	64.83	70.76	50.75	67.09	3
07	PB	72	60.03	69.83	71.06	69.8	53.01	61.42	6
11	PB	72	60.2	73.77	45.39	64.56	58.9	65.38	5
08	PB	76	73.32	77.08	72.49	68.33	73.96	69.08	1
12	PB	92	73.89	84.97	76.15	80.76	76.2	76.5	0
05	R	68	61.81	68.24	71.42	67.35	56.63	65.05	5
06	R	76	62.69	69.18	71.04	67.01	56.94	58.49	4
14	R	76	64.05	70.5	77.96	68.79	60.98	63.16	4
16	R	58	61.47	69.83	76.39	65.34	72.79	61.81	5
10	R	66	61.45	72.84	64.11	74.35	67.13	67.45	3
18	R	78	59.11	68.61	77.64	69.44	63.88	64.32	4
03	R	70	61.6	75.7	67.19	77.68	74.44	63.42	4
01	R	60	60.92	69.99	73.37	66.82	71.07	60.29	5
	**Mean**	**74.42**	**62.67**	**72.08**	**67.98**	**70.44**	**62.56**	**65.09**	
	**Minimum**	**58**	**56.43**	**64.94**	**45.39**	**59.59**	**50.75**	**57.16**	
	**Maximum**	**92**	**73.89**	**84.97**	**77.96**	**82.68**	**76.2**	**76.5**	
	**SD**	**8.63**	**4.22**	**4.95**	**8.65**	**5.54**	**8.64**	**4.82**	

Three QuIRC domains had wide variation in scores between PARC services: living environment; social interface; and treatments and interventions. Living environment was, on average, the highest-scoring domain across Victoria, with purpose-built services scoring higher (mean = 78, range 66–92) than those that had been converted (mean = 69, range 58–78). Treatments and interventions was the lowest-scoring domain across Victoria.

PARC service 12 scored highest for five of the seven domains and second highest on one domain. PARC service 4 scored second highest on two domains (self-management and autonomy, and recovery-based practice) and highest on another (human rights), but second lowest on social interface. PARC service 7 and PARC service 13 both scored below the state average on six out of seven domains. No service had the lowest score on more than one domain.

## Discussion

This paper provides the first insights into how adult sub-acute PARC services in Victoria operate and the support they deliver. Our data suggest that the majority are being run according to the government guidelines including localized variations (16). All service managers reported operating a partnership model and implementing the required policy and procedure documentation, although there was variation in terms of which service took the lead in policy development, reflecting local partnership arrangements, which are reflected in the sub-contracting arrangements developed by each AMHS. Further, there was variation between services in terms of the ratio of MHCSS staff and clinical staff, with some services having a much larger presence of clinical staff in the PARC service each day. It is possible that these differences may reflect variation in the balance between clinical and recovery-oriented functions and/or the different needs of the consumers accessing the service. While it is possible that these factors impact on consumer outcomes, further research to investigate this is required. Hence, the value of this mapping exercise in the context of our overall PARC services study is that these findings will assist us to interpret data from our other studies focused more on consumer outcomes.

A recovery-based model of service delivery was reported by all managers as the foundation of their PARC service delivery ethos. Aligned with the recovery-oriented model of care, a diverse range of group and individual programs were available to consumers; however, our data collection did not capture how the programs were delivered and to what extent consumers were able to direct the focus of the programs offered to meet their individual goals for recovery. For example, a group focusing on physical health is not recovery oriented if there is no choice, self-determination, or respect for individual decision making. Notwithstanding this limitation, the group programs described by participants indicated that most services provided programs covering the themes of *recovery and wellness*, *ADLs and self-management*, *physical health*, *psycho-therapeutic interventions*, *therapeutic milieu and activities*, and *social groups*. The combination of such a range of activities is aligned to the guidelines (16) and may support personal recovery by addressing the multi-faceted social, occupational, and health determinants of well-being and recovery. Concepts linked to recovery-oriented practice, such as connectedness, hope, identity, meaning and purpose, and empowerment (CHIME) (21), may be seen to be reflected in what is being supported by the activities in PARC services, for example, the involvement of peer workers in facilitating groups, connecting people to the local community, and support for self-management (22). These activities may also reflect the needs of people who attend PARC services and the sub-acute environment. Although there is an expectation that clinical services will also adopt recovery-oriented practice (23), this is much more difficult to achieve when inpatient length of stay is so short and the focus of care is generally on diagnosis, medication, and maintaining safety during a crisis (24). Hence, PARC services widen the opportunity to offer recovery-oriented group programs and other related activities. Further, the wide array of programs offered is likely to be a source of satisfaction for consumers who have reported dissatisfaction with the lack of engaging and meaningful activities in acute inpatient services (25).

Understanding how programs are offered and the extent of consumer input and choice requires further research. Just over half of the managers reported employing a peer worker, which may explain why not all PARC services reported individual peer support being available. Our survey did not specifically ask managers to describe how the mix of group and individual programs were decided; two managers chose to provide this detail, and their quotes provided strong indication of choice offered to consumers regarding the individual and group programs that they have access to during their admission.

The study also illustrated that consumers use the PARC services at different points in their recovery. They appeared to provide an important “bridging” service, acting as both a “step-up” service from community-based care, as well as a “step-down” service from inpatient care. The greater average number of consumers entering PARC services from the community is likely to be reflected in, and consequently shape, the types of activities and programs offered by the service. In their study of one Australian sub-acute residential service, Thomas and Rickwood (26) found that varying needs were identified by clients who were stepping up—seeking support with social skills and illness management—in contrast to those who were stepping down—valuing support with living skills and personal processes of recovery. Our findings appear to concur with those of Thomas and Rickwood (26), as the mix of consumers in PARC services may also explain the wide range of programs offered.

In terms of the KPIs set out in the government guidelines, the average length of stay was low, and the occupancy rate was highly variable. These findings pose a range of further questions, in particular, how a PARC service is positioned within a local system of care, with each local system operating under a range of unique forces that were not considered within this study. Length of stay is likely to be influenced by the relationship with the local inpatient unit and bed demand. The role of consumer preference in determining length of stay cannot be determined from these findings but may be an important factor. For example, in rural areas, the distance that consumers are from their home may deter extended stays (managers discussed this issue during the forum). The occupancy rates may be indicative of the length of time the PARC has been open, with newer services possibly still establishing themselves in the local area. It is worth noting that these figures came from the manager’s memory of the previous 6 months, and other studies in this program of work will access and analyze more rigorously collected state-wide data.

### QuIRC

The use of the QuIRC enabled valid comparison of the quality of the PARC services across Victoria and with similar English services. The individual domain average scores were generally higher than for supported accommodation services (27) in England. In the original validation of the QuIRC, service managers’ ratings of quality (as indicated by the domain scores) concurred with consumer ratings of their care and autonomy (17), allowing some confidence that the ratings reflect consumer views and experiences of the care provided. The living environment domain was the highest-scoring domain across the PARC services, indicating that the built environment was a particular strength in Victoria, particularly in the purpose-built services. The second-highest-scoring domain was self-management and autonomy, reflecting an emphasis on promoting consumers’ independence.

There was room for improvement on both the therapeutic environment domain, related to staffing, training, and supervision, and the treatments and interventions domain measuring clinical (medical, psychological, and social) interventions in PARC services. Further, there was a high degree of variation across PARC services on the treatments and interventions domain. PARC services have almost double the length of stay compared to inpatient units, but this remains a short length of stay when compared to other residential services. Hence, it may be challenging to provide tailored interventions efficiently, and this may explain the lower scores on the treatments and interventions domain than other domains. Further, these scores may highlight that although there is clinical input, PARC services are not operated as a clinical service. Usually, PARC services have more MHCSS resources compared to AMHS staffing. PARC services show comparable scores with similar service types in England but also variations within Victoria, demonstrating how the QuIRC can assist services to identify particular strengths and weaknesses (27). An example of a common challenge, suggested by low scores in both Victoria and England, is the incorporation of evidence-based practices into residential settings and mental health services in general (28).

The domain social interface (inclusion) was a mid-range score for the Victorian PARC services compared to scores on other domains, but in comparison to the supported accommodation services in the UK, it appears to be a strength for PARCs (27). Social interface measures the degree to which the service strengthens consumers’ social networks *via* making links with community resources and engages with the consumers’ families. This difference in PARC services may be due in part to the much shorter expected length of stay, thus producing a higher need to focus on external networks for consumers to ensure their continued recovery after discharge (15).

The domain of human rights was a relative strength for PARC services, highlighting that consumers’ legal and civil rights are promoted and that consumers are involved in decision making about their care. However, when compared with supported accommodation services (27), PARC services may have room for improvement. Achieving higher scores in relation to human rights may be an important indicator that PARC services are aligned with their stated principle of least possible restrictive practices. It may be that comparable improvement in this domain is difficult to achieve in a short-stay sub-acute environment, as compared to the English longer-stay supported accommodation services described in Killaspy et al. (27), but this could still represent an aspirational goal for PARC services.

Another potential area for improvement is the domain of recovery-based practice, even though scores in PARC services are on par with supported accommodation services in England (27). Previous research has found that recovery-oriented practice can be challenging to incorporate into bed-based services (29). Furthermore, PARC service consumers are likely to be attempting to avoid a hospital admission or have just had an acute admission. This sub-acute level of need may be maintaining a focus on clinical issues rather than personal recovery. Personal recovery has been defined as

“a deeply personal, unique process of changing one’s attitudes, values, feelings, goals, skills, and/or roles. It is a way of living a satisfying, hopeful, and contributing life even within the limitations caused by illness. Recovery involves the development of new meaning and purpose in one’s life as one grows beyond the catastrophic effects of mental illness (30; p2)”

and it may be that enabling emphasis on personal recovery is more challenging when there is a parallel imperative to achieve clinical outcomes—in particular, preventing admission (or readmission) to hospital. However, in English surveys, it has been found that higher scores related to recovery-based practice and human rights was positively associated with outcomes related to successful discharge to the community and progressing to more independent accommodation; hence, a challenge for PARC services may be to ensure that, in the context of a clinical and recovery-oriented partnership, the contributions of these domains to sub-acute care are appreciated (31).

### Strengths and Limitations

The state-wide scope and completeness of the data set are strengths of this study. A further strength is the inclusion of the QuIRC, a validated measure of service quality. There are tensions when developing a service delivery survey to accurately capture the government guidelines. In an effort to enhance accuracy, the Victorian survey was collaboratively developed with all stakeholders; however, there may be limitations in the survey’s ability to capture all of the activities that are occurring in the PARC services. Therefore, some important activities and how they are being delivered may not have been captured. The qualitative study included in our overall large evaluation project may enable more detailed description of the interventions in PARC services. Despite participants being asked to prepare relevant data before the forum, the findings may be limited by the recall of participants.

### Conclusions

Gathering comprehensive descriptions of 19 PARC services and their practice demonstrates the degree of variation in the structure, resourcing, and content and quality of care offered across the Victorian PARCs, and provides a contextual foundation for the more rigorous qualitative and quantitative studies that are in process. The findings indicate emerging evidence that PARCs are providing recovery-oriented services that offer consumers autonomy and social inclusion, which future studies may find links to a positive consumer experience. The range of individual and group programs is in line with the Victorian guidelines, offering practical assistance, therapeutic activities, and socialization opportunities which may provide an early indication of positive regard from consumers (8, 11, 15). However, current guidelines provide a framework only; PARC service variation comes from local interpretation, partnership arrangements, and the degree to which recovery principles and use of evidence-based practices are understood and employed by the partners. The QuIRC domains “worked” to describe the PARCs, with living environment and self-management and autonomy domains highlighting strengths and therapeutic environment and treatments and interventions relative weaknesses across the PARCs that might be explained by the variation in staffing, consumers, and context. Our findings regarding outcome measurement highlight the need for increased assessment of recovery outcomes. To date, PARC services have focused measurement of outcomes on satisfaction and experience of service type surveys; determining effectiveness of these services will be strengthened by the use of a range of other consumer outcome measures. Together, other studies in our broader evaluation of PARCs in Victoria will do more to see if there are differences in consumer outcomes across PARCs.

## Data Availability Statement

The datasets generated for this study are available on request to the corresponding author.

## Ethics Statement

The studies involving human participants were reviewed and approved by Ethics approval for this project was granted from The University of Melbourne’s Human Research Ethics Committee (project number: 1647880.1). The patients/participants provided their written informed consent to participate in this study.

## Author Contributions

CH, JF, LB, BH, and HK designed the study, with guidance from all other co-authors. JF, CH, LB, BH, and HK collected the data, with assistance from TH, LC, and PE. JF, CH, and LB analyzed the findings, with assistance from HK and BH. JF, CH, LB, BH, HK, TH, LC, and PE contributed to interpretation of the findings and reviewed and revised drafts of the manuscript.

## Funding

The project was funded by an National Health and Medical Research Council (NHMRC) partnership grant (APP1115907) and is a partnership between academic institutions, Mental Health Community Support Services (MHCSS), clinical mental health service providers, and the Victorian government.

## Conflict of Interest

The authors declare that the research was conducted in the absence of any commercial or financial relationships that could be construed as a potential conflict of interest.
